# Molecular Reclassification of Diffuse Gliomas: A Feasibility Study of Clinical Features and Survival Outcomes in Colombia

**DOI:** 10.3390/cancers18203364

**Published:** 2026-10-17

**Authors:** Edgar G. Ordóñez-Rubiano, Fabiana Rodríguez, Cristian F. Siabato-Vargas, Luz Dary Gutiérrez-Castañeda, Nicolás Rincón-Arias, Sebastián Espinosa, Alba Cómbita, Matías Baldoncini, Diego F. Gómez-Amarillo, Fernando Hakim, Pedro Andrade-Andrade, César Payán-Gómez, Oscar F. Zorro-Guío, Javier G. Patiño-Gómez, Irene Riveros-Barrera, Oscar Mendoza, Rafael Parra-Medina

**Affiliations:** 1 School of Medicine, Universidad Nacional de Colombia, Bogotá 111321, Colombia; 2 Department of Neurosurgery, Universidad FUCS, Hospital de San José—Sociedad de Cirugía de Bogotá, Bogotá 111411, Colombia; 3 Department of Neurosurgery, Hospital Universitario Fundación Santa Fe de Bogotá, Bogotá 110111, Colombia; 4 Department of Pathology, Universidad FUCS, Hospital de San José—Sociedad de Cirugía de Bogotá, Bogotá 111411, Colombia; 5 Group of Basic Sciences in Health, Universidad FUCS, Hospital Infantil Universitario de San José, Bogotá 111221, Colombia; 6 Department of Microbiology, Universidad Nacional de Colombia, Bogotá 111321, Colombia; 7 Department of Neurosurgery, San Fernando Hospital, San Fernando B1646, Buenos Aires, Argentina; 8 School of Medicine, Universidad de Los Andes, Bogotá 111711, Colombia; 9 Dirección Académica, Universidad Nacional de Colombia, Sede de La Paz, La Paz 202010, Colombia; 10 Department of Pathology, Instituto Nacional de Cancerología, Bogotá 111511, Colombia; 11 Research Institute, Universidad FUCS, Hospital de San José—Sociedad de Cirugía de Bogotá, Bogotá 111411, Colombia

**Keywords:** glioma, classification, World Health Organization, glioblastoma, oligodendroglioma, astrocytoma

## Abstract

**Background/Objectives:** The 2021 World Health Organization Classification of Tumors of the Central Nervous System (WHO CNS5) integrates histological and molecular criteria for adult diffuse gliomas, but comprehensive molecular testing may be difficult to implement in resource-limited settings. This study assessed the feasibility of a sequential, marker-based approach for diagnostic reassessment and described its association with clinical characteristics and overall survival (OS) in adults from Colombia. **Methods:** We retrospectively analyzed 68 adults with diffuse gliomas treated at two referral hospitals in Bogotá, Colombia, between 2015 and 2023. Molecular assessment used IHC, FISH, PCR, and Sanger sequencing according to the available tissue and diagnostic workflow. The study did not include comprehensive NGS or systematic assessment of combined whole-chromosome 7 gain/chromosome 10 loss (+7/−10). **Results:** The diagnosis changed after molecular reassessment in 51.5% of cases. The proportion classified as glioblastoma increased from 48.5% under the historical histologic classification to 66.2% under the WHO CNS5-based algorithm, whereas FISH-defined oligodendrogliomas decreased from 17.6% to 11.8%. OS differed among historical histologic groups (*p* = 0.03), but not among the WHO CNS5-based groups (*p* = 0.13). Female sex was associated with longer OS in unadjusted analysis (*p* = 0.02), while age was the only independent predictor in the multivariable analysis. **Conclusions:** Sequential molecular reassessment materially changed diagnostic categorization in this cohort and was feasible using locally available marker-based methods. However, the results are exploratory, limited by small molecular subgroups, incomplete molecular coverage, and the retrospective treatment context; they should not be interpreted as demonstrating improved treatment selection or survival after reclassification.

## Simple Summary

This study evaluated the feasibility of applying the 2021 World Health Organization Classification of Tumors of the Central Nervous System (WHO CNS5) to adult diffuse gliomas in Colombia. We retrospectively analyzed 68 patients treated at two referral hospitals in Bogotá between 2015 and 2023. A sequential molecular workflow using immunohistochemistry (IHC), fluorescence in situ hybridization (FISH), polymerase chain reaction (PCR), and Sanger sequencing was used to support diagnostic reassessment. The diagnosis changed in 51.5% of cases, particularly among tumors initially classified as astrocytoma or glioma, not otherwise specified (NOS). The proportion classified as glioblastoma increased from 48.5% under the historical histologic classification to 66.2% under the study’s WHO CNS5-based algorithm, while FISH-defined oligodendrogliomas decreased from 17.6% to 11.8%. Overall survival differed across historical histologic groups (*p* = 0.03), whereas differences across the WHO CNS5-based groups were not statistically significant (*p* = 0.13). Female sex was associated with longer survival in unadjusted analysis, while age was the only independent predictor in the multivariable model. Because comprehensive sequencing and systematic +7/−10 testing were not performed, these findings should be interpreted as an exploratory assessment of diagnostic feasibility rather than complete molecular ascertainment or evidence of treatment benefit.

## 1. Introduction

Adult diffuse gliomas are infiltrative central nervous system (CNS) tumors with substantial biological and clinical heterogeneity [1,2]. The 5th edition of the World Health Organization (WHO) Classification of Tumors of the Central Nervous System (WHO CNS5) introduced an integrated molecular and histological framework that defines three major adult-type diffuse gliomas: astrocytoma, isocitrate dehydrogenase (IDH)-mutant (IDHmut); oligodendroglioma, IDHmut and 1p/19q-codeleted; and glioblastoma (GBM), IDH-wildtype (IDHwt) [3]. This approach reduces reliance on morphology alone [4,5,6] and incorporates molecular alterations that have direct diagnostic and grading implications [3]. Importantly, the WHO CNS5 framework also requires careful distinction between molecularly confirmed alterations and immunohistochemical surrogate findings, particularly for IDH, ATRX, and TP53. These changes have increased diagnostic precision but also created implementation challenges in settings where comprehensive molecular profiling is not routinely available [7,8].

**Figure 1:**
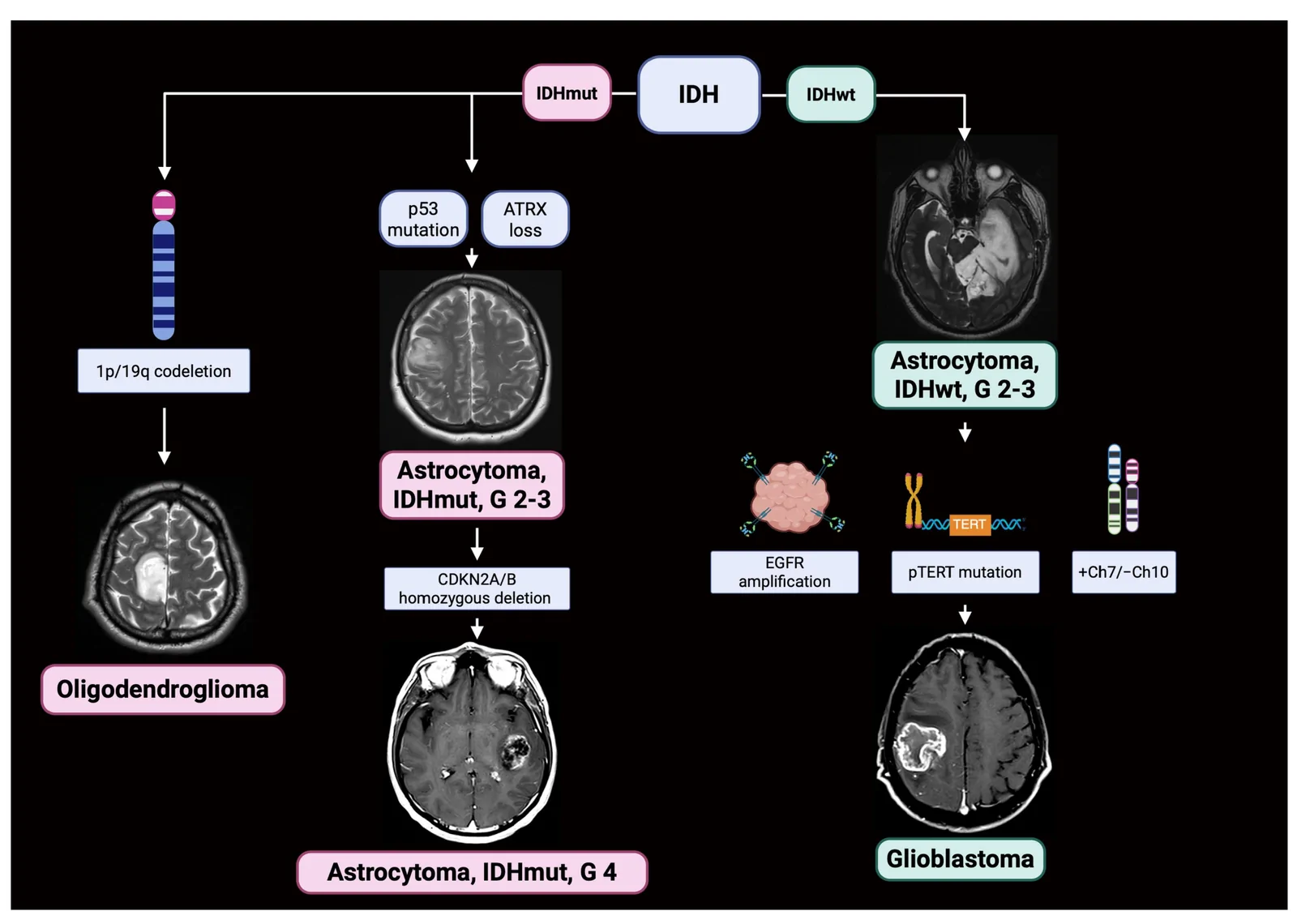
Schematic overview of molecular features used in the WHO CNS5-based study algorithm for adult diffuse gliomas. ATRX loss and abnormal p53 staining represent immunohistochemical surrogate findings rather than genetically confirmed mutations. +7/−10 is shown as a WHO CNS5 criterion but was not systematically assessed in this cohort. Created in BioRender. Ordonez-Rubiano, E. (2026). https://BioRender.com/m7bft8g.

Molecular findings relevant to adult diffuse glioma classification include IDH1/IDH2 mutation status, whole-arm 1p/19q codeletion, ATRX and TP53 alterations, CDKN2A/B homozygous deletion in selected IDHmut astrocytomas, TERT promoter (pTERT) mutation, EGFR amplification, and combined whole-chromosome 7 gain/chromosome 10 loss (+7/−10) in appropriate IDHwt tumors (Figure 1) [3,9,10].

These alterations should not be treated as interchangeable: ATRX loss and abnormal p53 staining are protein-level surrogate findings, whereas deletion, amplification, mutation, and copy-number changes represent distinct molecular events. These molecular alterations enhance prognostic stratification and inform therapeutic decisions, enabling the use of more specific and targeted treatments [11,12]. Also, IDHmut status and 1p/19q codeletion define oligodendroglioma [13], whereas pTERT mutation, EGFR amplification, or +7/−10 may support a diagnosis of GBM, IDHwt, in the appropriate histological and molecular context [3]. Molecular information can also influence therapeutic considerations, including the use of IDH-directed therapies in selected IDHmut tumors [14], although the present study was not designed to evaluate treatment selection or treatment response.

Comprehensive molecular profiling remains difficult to implement in many low- and middle-income countries (LMICs) because of infrastructure, personnel, and cost constraints [15,16,17,18]. Marker-based methods such as immunohistochemistry (IHC), FISH, PCR, and Sanger sequencing can provide targeted information when next-generation sequencing (NGS) is unavailable, but their limitations must be explicitly recognized, particularly for noncanonical IDH alterations, whole-arm copy-number events, gene fusions, and other complex genomic changes. The present study therefore evaluates a pragmatic sequential workflow rather than proposing it as an equivalent substitute for comprehensive sequencing. In addition to molecular classification, clinical and radiological information remains important in longitudinal glioma care; in selected postoperative scenarios, adjunctive imaging (e.g., PET) may help distinguish recurrent tumor from postoperative granulomatous change [19]. The objective of this study was to assess of implementing a sequential molecular reassessment workflow and describe its effects on diagnostic categorization, clinical features, and overall survival (OS) in adult patients from Colombia.

## 2. Materials and Methods

This multicenter retrospective cohort study included adults (≥18 years) with diffuse gliomas treated at Hospital de San José—Sociedad de Cirugía de Bogotá and Hospital Infantil Universitario de San José, Bogotá D.C., Colombia, between January 2015 and December 2023. Eligible participants underwent maximal safe resection or intentional biopsy and had clinical records and tumor tissue available for the molecular reassessment performed in this study. Of 261 screened records, 68 met the study eligibility criteria. Because the source records did not contain a standardized prospective screening log, detailed reasons for exclusion among the remaining 193 screened records could not be reconstructed retrospectively and are therefore identified as a limitation rather than inferred.

The electronic records of both institutions were reviewed to collect demographic and clinical data, including the original histopathological diagnosis, molecular and immunohistochemical findings, radiological characteristics, presenting symptoms, tumor location, pre- and postoperative Karnofsky Performance Score (KPS), extent of resection (EOR), postoperative complications, follow-up, and OS. For the comparative analysis, we harmonized original diagnoses into broad historical histologic categories (oligodendroglioma, astrocytoma, glioma NOS, and GBM) [4] rather than assuming every patient was diagnosed under the same WHO edition during 2015–2023. We then performed molecular reassessment using the available data and interpreted it against WHO CNS5 criteria [3]. The study reassessment occurred after the patients had completed their original surgical and adjuvant treatment and therefore did not influence treatment selection. EOR was categorized as gross-total resection (GTR; complete radiological resection), near-total resection (NTR; >90% resection), subtotal resection (STR; <90% resection), or intentional biopsy. For GBM cases, EOR was assessed using the enhancing component; for grade 2–3 gliomas, it was assessed using the T2/FLAIR abnormality. Vital status was verified using national databases from the Departamento Administrativo Nacional de Estadística (DANE) and the Registraduría Nacional del Estado Civil of Colombia.

### 2.1. Informed Consent Statement

The Ethics Committee and Institutional Review Board of the Fundación Universitaria de Ciencias de la Salud—FUCS waived patient consent due to the retrospective nature of this study, which had no impact on patient treatment or outcomes. There is no identifiable information about the patients in this manuscript.

### 2.2. Molecular Profiling Protocol

Molecular reassessment was performed using a sequential marker-based protocol selected according to the available tissue and the diagnostic question. The assays included IDH1 R132H IHC; ATRX and p53 IHC as surrogate protein-level assessments; FISH for 1p/19q and CDKN2A/B; EGFR amplification by FISH; PCR-based assessment of pTERT; and bidirectional Sanger sequencing of the amplified TERT fragment. This workflow was intended to support an integrated diagnosis using locally available techniques. It was not equivalent to comprehensive NGS and did not include a systematic assay for combined whole-chromosome +7/−10. Accordingly, the resulting diagnoses should be interpreted as WHO CNS5-based assignments supported by the available data rather than complete molecular ascertainment of every WHO CNS5 criterion [3].

#### 2.2.1. IDH1, TP53, and ATRX Assessment

Formalin-fixed, paraffin-embedded (FFPE) tissue samples from patients were used. Tissue sections, with a thickness of 4 μm, were mounted on slides and left to dry overnight at 37 °C. For deparaffinization, incubations were performed in a drying oven at 60 °C for 1 h, followed by immersion in xylene and rehydration in ethanol.

Heterozygous missense mutations affecting codon 132 of IDH1 are common in gliomas, with R132H representing the most frequent variant [20,21]. We therefore used the anti-IDH1 R132H primary antibody (clone H09; Roche^®^, Basel, Switzerland) with the UltraView Universal DAB detection kit (Roche^®^). Antigen retrieval was performed in nitrate buffer (pH 6.0) at 95 °C for 20 min, followed by blocking, primary antibody incubation, DAB detection, and hematoxylin counterstaining on a Ventana BenchMark ULTRA device (Roche, Grenzach-Wyhlen, BW, Germany). Examination under light microscopy identified the expected R132H staining pattern. Positive staining was interpreted as evidence of an IDH1 R132H mutation. Importantly, negative R132H IHC does not exclude noncanonical IDH1 or IDH2 mutations. The available study workflow did not include systematic reflex sequencing of R132H-negative cases; therefore, such cases were treated as IDHwt within the study algorithm, and this potential misclassification is explicitly recognized as a limitation.

ATRX expression was assessed by immunohistochemistry using a polyclonal anti-ATRX antibody (Vitro®, Basel, Switzerland, catalog no. DV200-0056). Antigen retrieval was performed using citrate buffer (pH 6.0) at 95 °C for 20 min, followed by incubation with the primary antibody and visualization using the Vitro® DAB detection system. ATRX expression was evaluated by light microscopy. Loss of ATRX expression was defined as complete absence of nuclear staining in tumor cells in the presence of retained nuclear staining in non-neoplastic cells, including endothelial, inflammatory, or other stromal cells, which served as internal positive controls. Cases lacking adequate internal positive controls were considered non-interpretable. Preserved nuclear staining in tumor cells was classified as retained ATRX expression.

We assessed p53 expression by immunohistochemistry using the monoclonal anti-p53 antibody (clone DO-7, Roche Diagnostics), following the same general immunohistochemical workflow. We evaluated nuclear staining in tumor cells for distribution and intensity. An abnormal (mutant-pattern) p53 staining pattern was defined as either diffuse strong nuclear overexpression in tumor cells or complete absence of nuclear staining (null pattern) in the presence of appropriate internal positive controls. A heterogeneous pattern of nuclear staining with variable intensity was considered a wild-type staining pattern. p53 immunohistochemistry was used as a surrogate marker for TP53 alteration for reclassification purposes in IDHmut, 1p/19q-non-codeleted tumors.

#### 2.2.2. Fluorescence In Situ Hybridization Analysis of 1p/19q Codeletion and CDKN2A/B Homozygous Deletion

For 1p/19q codeletion analysis, only IDHmut glioma samples underwent fluorescence in situ hybridization (FISH). FFPE sections were dried at 90 °C for 2 h, then deparaffinized in xylene (4 × 10 min) and rehydrated in absolute ethanol (2 × 5 min), followed by air-drying. Pretreatment involved immersing the slides in a preheated solution at 90 °C for 4 h, followed by rinsing in distilled water for 4 min and air-drying. Pepsin (100 µL) was then applied at 37 °C for 12–15 min. Slides were subsequently dehydrated through graded ethanol and air-dried.

Hybridization was performed using 10 µL of Hybridization Buffer combined with 1 µL of the corresponding Abbott Vysis^®^ probe. The hybridization mixture was applied to the target area, sealed, denatured at 80 °C for 10 min, and incubated overnight at 37 °C. Following hybridization, slides were washed with 0.4× saline-sodium citrate (SSC)/Tween at room temperature for 5 min and subsequently with 2× SSC at 72 °C for 2 min. DAPI was used as a counterstain, and slides were evaluated by fluorescence microscopy.

The Vysis LSI 1p36 SpectrumOrange/1q25 SpectrumGreen and Vysis LSI 19q13 SpectrumOrange/19p13 SpectrumGreen probes (Abbott Molecular Inc., Des Plaines, IL, USA) were used to assess 1p36 and 19q13 deletion status, respectively. For each sample, 100 independent nuclei were evaluated for 1p36/1q25 and an additional 100 independent nuclei for 19q13/19p13. Deletion status was determined based on the ratio between the number of signals corresponding to the region of interest (red) and the corresponding internal control region (green). A ratio < 0.83 was considered indicative of 1p36 deletion, whereas a ratio < 0.88 was considered indicative of 19q13 deletion, according to established criteria. When the calculated ratio was exactly equal to the respective cutoff after evaluation of 100 nuclei, additional nuclei were analyzed to clarify the result.

The genomic regions assessed were limited to those targeted by the probes used. The Vysis LSI 1p36 probe covers approximately 435 kb and includes the TP73 and MEGF6 genes, extending telomerically beyond the MEGF6 locus. The Vysis LSI 19q13 probe covers approximately 380 kb and includes sequences encompassing CRX, GLTSCR1, GLTSCR2, and ANGPTL1, extending to the centromeric region of the GLTSCR1 locus. Accordingly, FISH findings were interpreted as reflecting the status of the specific genomic regions targeted by these probes and were not considered representative of deletion across the entire 1p or 19q chromosomal arms. IDHmut gliomas demonstrating the predefined 1p/19q FISH deletion pattern were categorized as FISH-defined oligodendroglioma within the study algorithm, recognizing that locus-specific FISH does not directly establish whole-arm 1p/19q codeletion.

On the other hand, CDKN2A/B deletion status was assessed by FISH using the dual-color Vysis LSI CDKN2A/CEP 9 probe (Abbott Molecular Inc. ^®^). For each hybridized sample, 100 independent tumor nuclei were evaluated. The LSI CDKN2A probe spans approximately 222 kb and targets the 9p21 region, encompassing the CDKN2A and CDKN2B loci and additional genomic sequences. The CDKN2A target region is labeled with SpectrumOrange, whereas the chromosome 9 centromeric probe (CEP 9), used as an internal control, is labeled with SpectrumGreen.

Deletion status was assessed based on the proportion of tumor nuclei showing loss of the CDKN2A target signal and the ratio of target to control signals. A cutoff of ≥20% of tumor nuclei showing loss of the target signal and a red-to-green signal ratio < 0.80 was used to define deletion.

The zygosity of the deletion was determined from the signal pattern in individual nuclei. A heterozygous deletion was defined by two green CEP 9 signals and one red CDKN2A signal, whereas a homozygous deletion was defined by two green CEP 9 signals with complete absence of red CDKN2A signals. Only cases demonstrating a homozygous deletion pattern were classified as having a CDKN2A/B homozygous deletion for molecular classification purposes. Cases showing a heterozygous deletion pattern were not considered positive for CDKN2A/B homozygous deletion. The IDHmut grade 2–3 astrocytomas were consequently classified as IDHmut astrocytoma grade 4 in a case-by-case grading.

#### 2.2.3. EGFR Amplification

To assess EGFR amplification and pTERT gene mutations, we selected samples from patients with IDHwt grade 2–3 astrocytomas and GBMs. EGFR fluorescence in situ hybridization (FISH) was performed on 2 µm formalin-fixed, paraffin-embedded (FFPE) tissue sections using the CytoCell LPS003-A dual-color EGFR amplification probe (Cytocell), Oxford Gene Technology IP Limited, Kidlington, Oxfordshire, UK. The probe targets the EGFR locus at 7p11.2 with a SpectrumOrange-labeled probe of approximately 295 kb and the chromosome 7 centromeric region (D7Z1/CEP7; 7p11.1–q11.1) with a SpectrumGreen-labeled control probe. FISH was performed according to the manufacturer’s instructions with minor modifications. Briefly, tissue sections were deparaffinized using heat treatment at 100 °C and xylene, rehydrated through graded ethanol, and subjected to heat-induced pretreatment using either 98 °C for 30 min or citrate buffer (pH 6.0) at 80 °C for 1 h. Enzymatic digestion was subsequently performed with pepsin at 37 °C for 8 min, followed by washing in 2× saline-sodium citrate (SSC), dehydration, and air drying. Subsequently, 8 µL of probe was applied to each slide, followed by co-denaturation at 85 °C for 5 min and hybridization at 37 °C for 48 h. Post-hybridization washes were performed in 0.4× SSC at 74 °C for 2 min and 2× SSC at room temperature for 30 s. Slides were counterstained with DAPI and analyzed by fluorescence microscopy, LEICA DM 2500 microscope (Leica, Wetzlar, Germany).

At least 10 non-overlapping, morphologically intact tumor nuclei were evaluated per section. EGFR amplification was assessed using the ratio of EGFR to CEP7 signals at the nuclear level. A case was classified as EGFR-amplified when >50% of the evaluated tumor nuclei demonstrated an EGFR/CEP7 signal ratio > 2.0. This >50% nuclear threshold was based on the criteria proposed by French et al., derived from an analysis of more than 1000 GBM samples [22]. Cases with an EGFR/CEP7 ratio < 2.0 were classified as non-amplified. Cases showing increased CEP7 copy number without a corresponding focal increase in the EGFR/CEP7 ratio were interpreted as chromosome 7 polysomy rather than focal EGFR amplification.

The CytoCell LPS003-A probe is classified by the manufacturer as an Analyte Specific Reagent (ASR), for which analytical and performance characteristics are not established by the manufacturer. Therefore, the criteria used to define EGFR amplification were based on peer-reviewed literature rather than solely on the manufacturer’s product specifications. No cases demonstrated heterogeneous or borderline amplification patterns requiring additional interpretative criteria.

#### 2.2.4. pTERT Mutation

Mutations in pTERT were identified using endpoint PCR with the primers Forward: 5′-AGTGGATTCGCGGGCACAGA-3′ and Reverse: 5′-CAGCGCTGCCTGAAACTC-3′. PCR amplification was performed on 50–100 ng of genomic DNA using the AmpliTaq Gold 360 Master Mix (Thermo Fisher Scientific^®^, Waltham, MA, USA) in 20 μL reactions containing 0.3 μM primers. Thermal cycling was conducted on an Applied Biosystems StepOnePlus™ System (v2.3) under the following conditions: 95 °C for 10 min, followed by 35 cycles of 95 °C, 63 °C, and 72 °C, with a final extension at 72 °C for 7 min.

Amplification products were verified by 1% agarose gel electrophoresis stained with SYBR™ Safe and visualized on a Gel Doc™ XR+ System (Bio-Rad^®^, Hercules, CA, USA). The target pTERT fragment was purified from the gel using the Monarch^®^ DNA Cleanup and Gel Extraction Kit (New England Biolabs^®^, Ipswich, MA, USA) and eluted in 10 μL of nuclease-free water. Purified amplicons were Sanger-sequenced bidirectionally, and chromatograms were analyzed using NovoSNP v3.0.1 software.

### 2.3. Genomic DNA Extraction

We extracted genomic DNA from FFPE tissue samples in multiple phases using PureLink™, Rheine, Germany, Genomic DNA Mini Kit and blood DNA extraction kits. Initially, pieces of the tissue block were produced using a microtome, with a standard thickness of 10–60 µm based on the pathologist’s recommendations. The first parts were eliminated to prevent contamination due to air exposure. The slices were then immediately placed in microcentrifuge tubes and deparaffinized with xylene, followed by ethanol to remove any residue, ensuring that the tissue was free of paraffin and other impurities. The DNA was purified using PureLink™ Genomic DNA Mini Kit (REF K1820-01; Life technologies Corp. Carlsbad, CA, USA), washed with buffers AW1 and AW2 to eliminate any leftover contaminants, then eluted in Buffer ATE to yield high-quality DNA appropriate for future molecular applications. This procedure was used to ensure the efficient extraction and stringent purification of DNA from FFPE tissue, which is required for deep genetic and molecular studies.

### 2.4. Statistical Analysis

Variables were classified as continuous, binary categorical, or nominal categorical. Categorical variables were compared using χ^2^ or two-sided Fisher’s exact tests, as appropriate. For tables larger than 2 × 2, Pearson’s χ^2^ test with Yates’ continuity correction was used when appropriate; when ≥1 expected cell count was <5, Monte Carlo simulation with fixed margins was applied (seed = 2025). Continuous variables were summarized as mean ± SD and median (IQR) and compared using the Kruskal–Wallis test; paired preoperative and postoperative measurements were compared using the Wilcoxon signed-rank test. KPS was recorded in 10-point increments on the 0–100 scale; values of 0 and 5 were treated as missing-data codes rather than valid scores because a preoperative KPS of 0 was incompatible with the corresponding postoperative data.

OS was calculated from surgery to death or last documented follow-up, with follow-up completed in December 2023. Vital status was verified through official DANE and Registraduría Nacional del Estado Civil records. Patients alive at the end of follow-up were censored at their last documented follow-up. Survival analyses were considered exploratory because of the small molecular subgroups, limited event counts, and retrospective diagnostic reassignment after treatment. All tests were two-sided with α = 0.05; missing data were handled by available-case analysis, and no multiplicity adjustment was applied unless otherwise stated. Treatment-related variables, including radiotherapy, temozolomide, treatment completion, and subsequent therapies, were inconsistently available and were therefore excluded from survival and adjusted analyses.

A multivariable Cox proportional hazards model was prespecified using four clinically selected covariates: age at diagnosis (per year), preoperative KPS (per point), extent of resection (GTR/NTR vs. STR/biopsy), and WHO CNS5 tumor subtype. No stepwise or significance-based variable selection was performed. All 68 patients had complete data for these covariates and were included in the model. The proportional hazards assumption was assessed using scaled Schoenfeld residuals, both for individual covariates and globally; variables violating the assumption were incorporated as stratification factors in a sensitivity analysis. Analyses were performed in R 4.5.2 (survival, survminer) and Python 3.x (pandas, scipy).

## 3. Results

**Figure 2:**
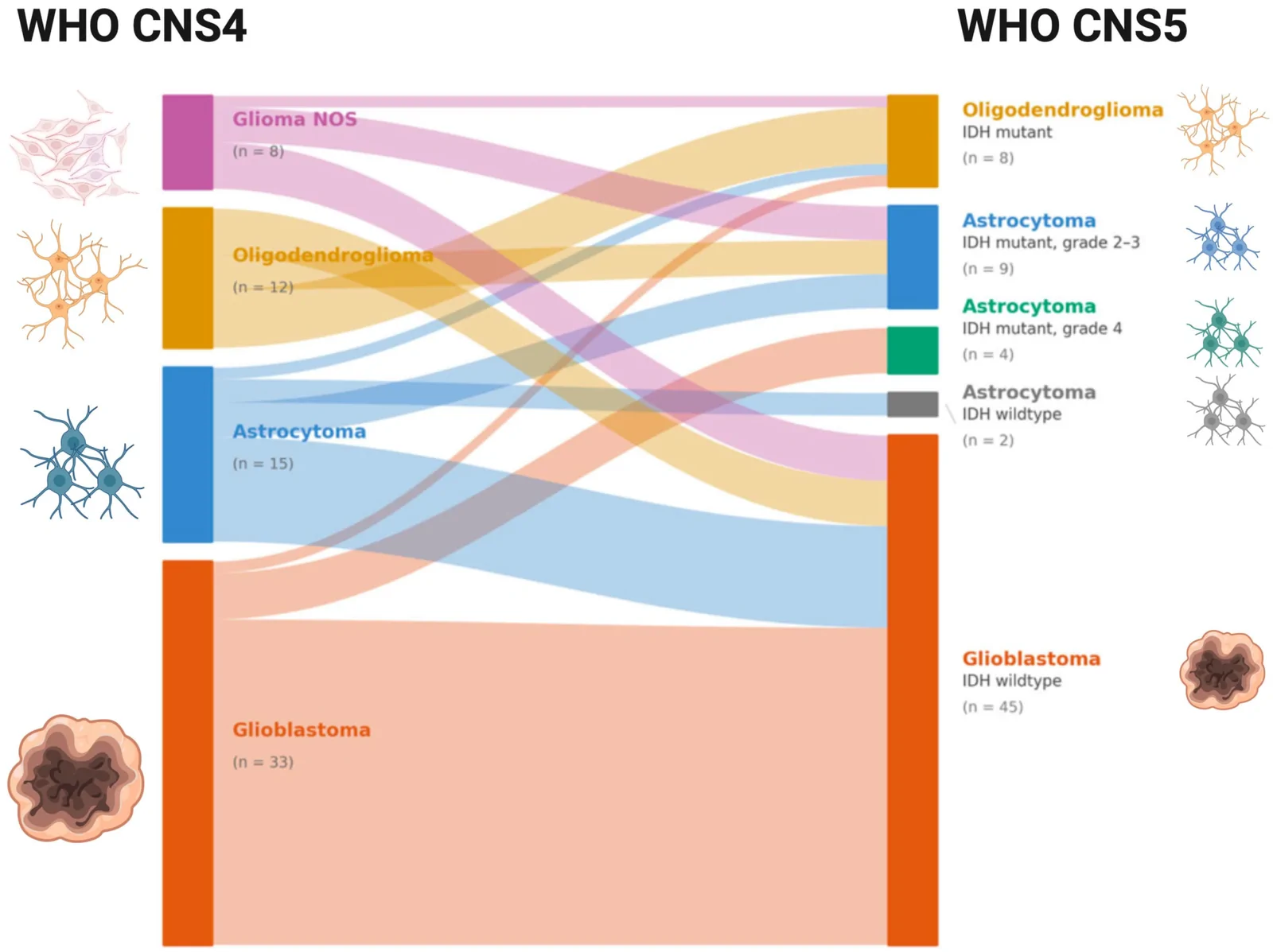
A Sankey diagram demonstrating the reclassification of diffuse gliomas from WHO CNS4 to WHO CNS5. Created in BioRender. Ordonez-rubiano, E. G. (2026) https://BioRender.com/3aed9wd.

Of 261 screened patients, 68 met the inclusion criteria and were analyzed. The cohort comprised 40 (58.9%) men and 28 (41.2%) women, with a mean age at diagnosis of 56.6 ± 15.5 years (range: 18–80). According to the WHO CNS5, the reclassification was 66.2% GBM, 13.2% astrocytoma, IDHmut (grades 2–3), 11.8% FISH-defined ODG (grades 2–3), 5.9% astrocytoma, IDHmut (grade 4), and 2.9% astrocytoma, IDHwt (grades 2–3) (Table 1). A total of 51.5% of the cases changed in the diagnosis after molecular reclassification, primarily those initially categorized as astrocytoma or glioma, NOS (Figure 2 and Table 2). The two tumors classified as IDHwt astrocytoma, grades 2–3, lacked the molecular features assessed in this study that would support assignment as GBM, IDHwt; however, because +7/−10 was not systematically evaluated and comprehensive molecular profiling was not performed, these assignments should be regarded as provisional.

Clinical characteristics did not differ significantly across the WHO CNS5-based groups for the variables reported in Table 1. Tumors were most frequently located in the frontal (30.9%) and temporal (25.0%) lobes (*p* = 0.971). Sensory deficits (44.1%) and motor deficits (41.2%) were the most frequent presenting symptoms (*p* = 0.207). TP53 and ATRX were assessed as part of the WHO CNS5-based diagnostic workflow; however, neither finding was determinative of diagnostic reassignment in this cohort. Mean preoperative KPS scores were 95.7 ± 5.3 for FISH-defined ODGs, 85.0 ± 15.2 for astrocytomas IDHmut (grades 2–3), 82.5. ± 15.0 for astrocytomas IDHmut (grade 4), 95.0 ± 7.1 for astrocytomas IDHwt, and 92.0 ± 14.2 for GBMs, with postoperative values showing parallel trends (94.3 ± 5.3, 65.0 ± 23.5, 70.0 ± 23.1, 75.0 ± 35.4, 86.2 ± 13.1, respectively) (Table 1).

GTR or NTR was achieved in 35 of 68 cases (51.5%). GTR was achieved in 8 cases (11.8%) and NTR in 27 cases (39.7%) (*p* = 0.217). New postoperative motor deficits occurred in 18 patients (26.5%), with full recovery within three months in 94.4% of these cases. Mean follow-up was 24.4 ± 26.7 months.

**Figure 3:**
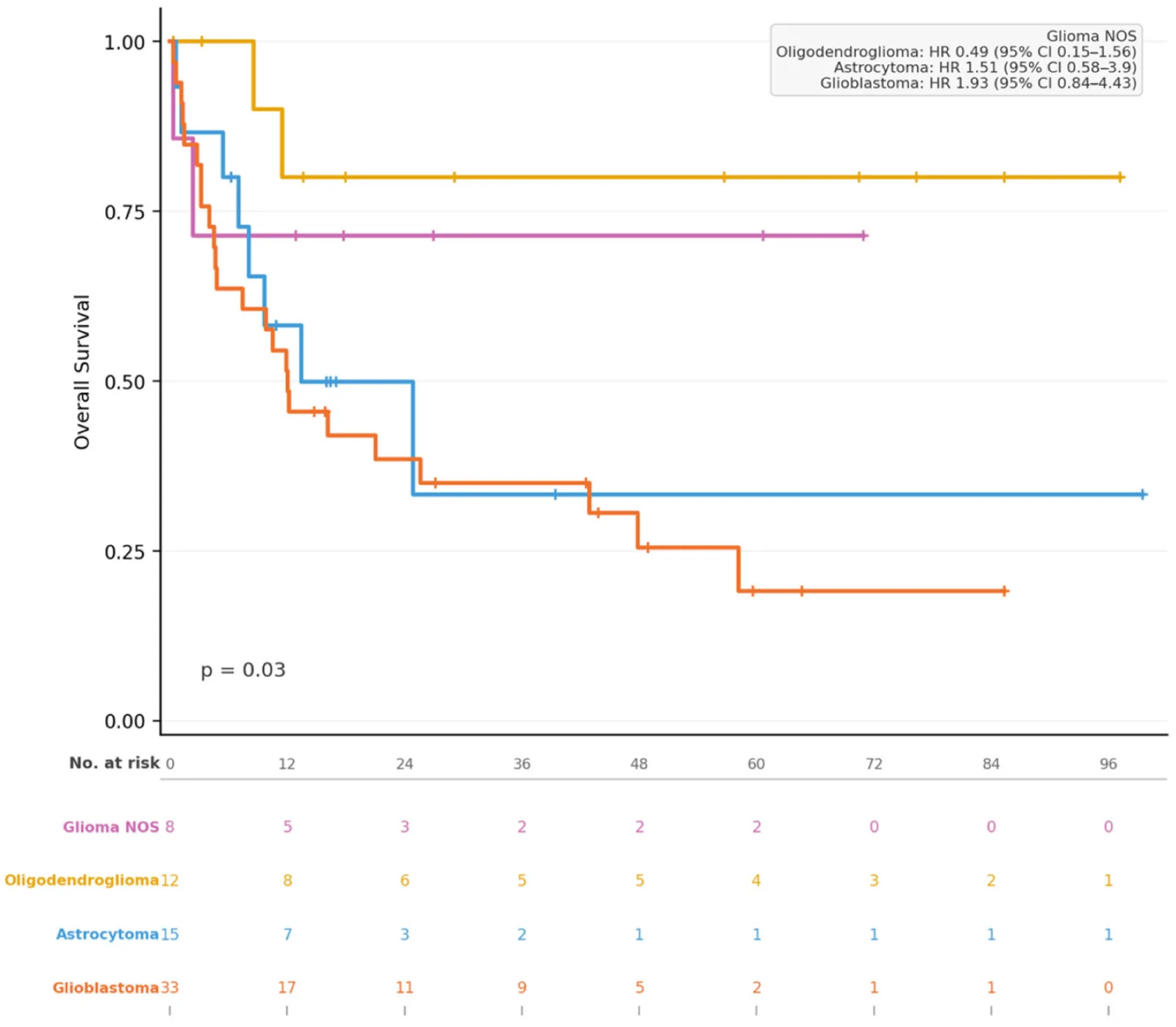
Kaplan–Meier overall survival curves according to the historical histologic classification (WHO CNS4). The global comparison was statistically significant (*p* = 0.03); however, the analysis is retrospective and reflects historical diagnostic categories.

**Figure 4:**
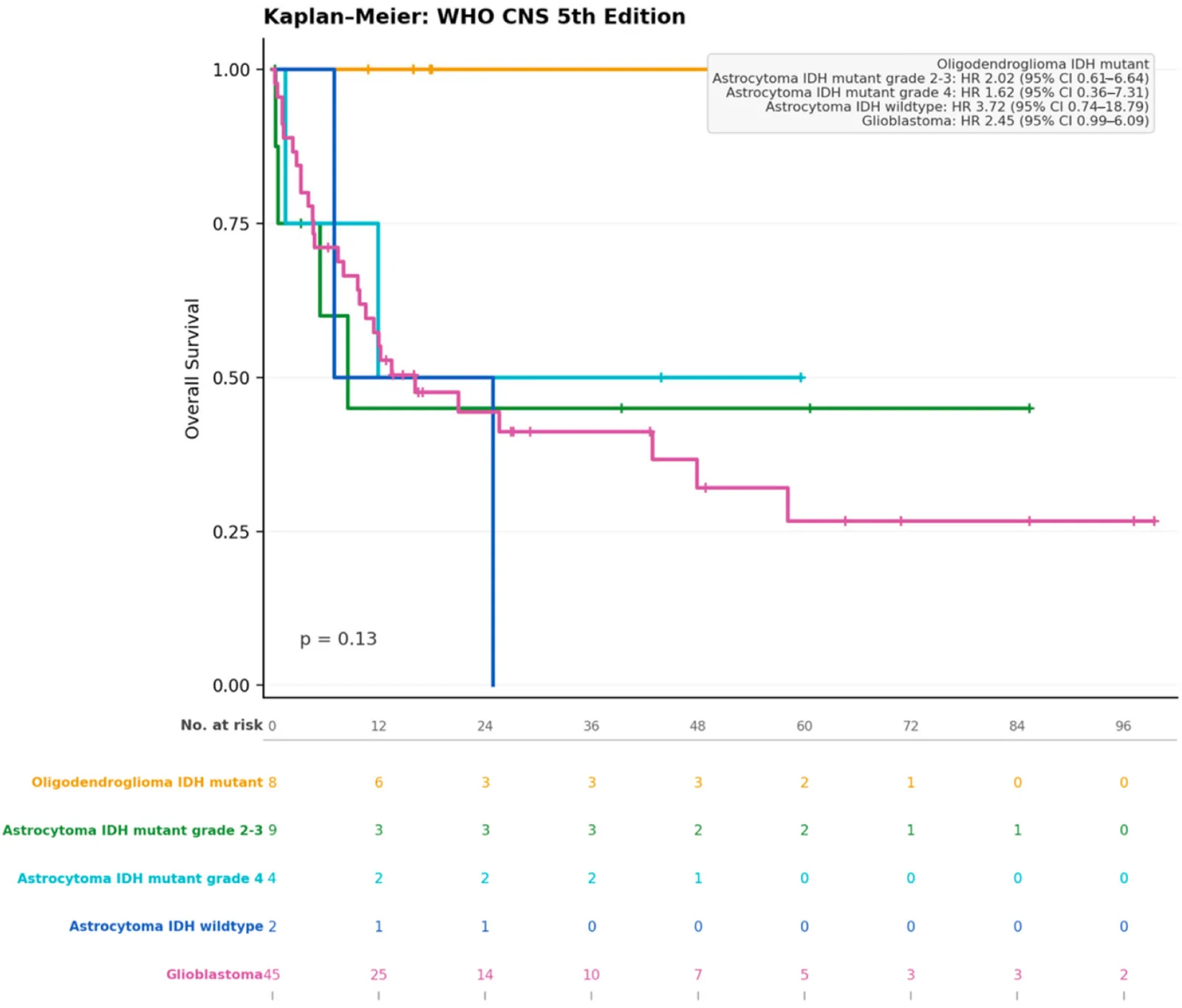
Kaplan–Meier overall survival curves according to the WHO CNS5-based study algorithm. The global comparison was not statistically significant (*p* = 0.13), and several molecular subgroups were small.

**Figure 5:**
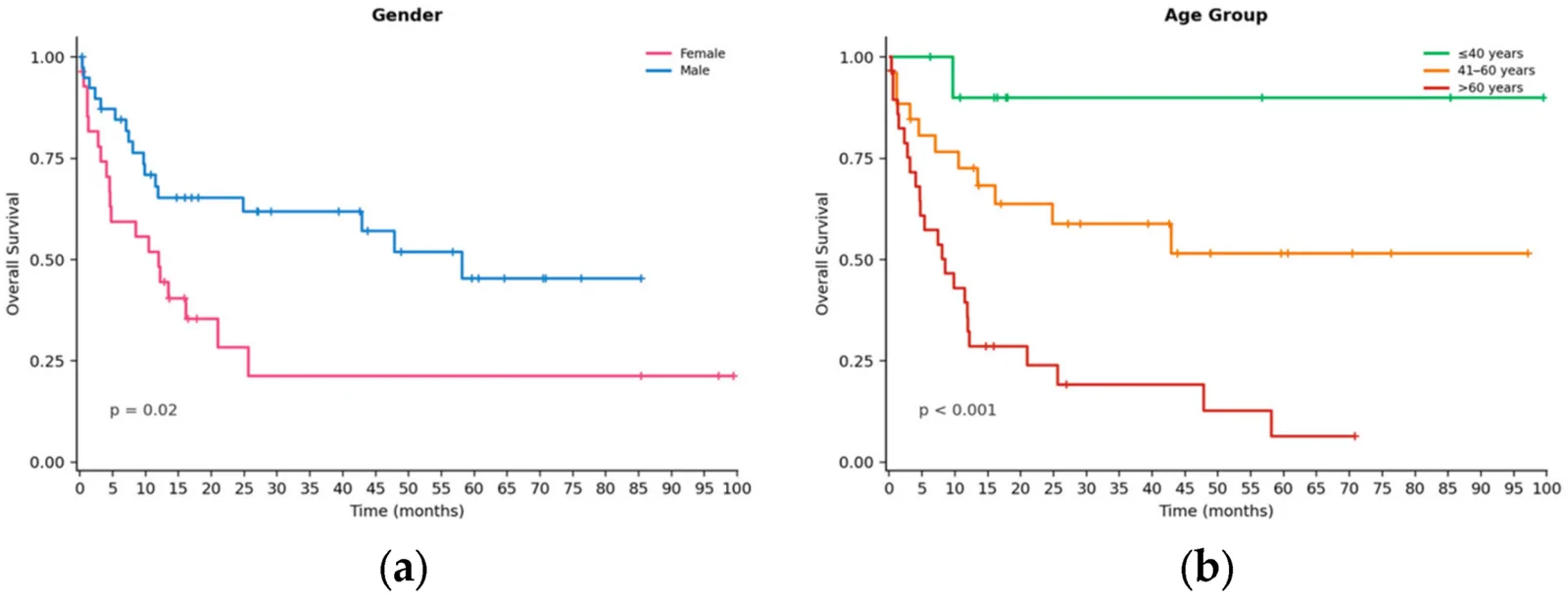
Kaplan–Meier overall survival curves comparing (**a**) female and male patients (unadjusted *p* = 0.02) and (**b**) age groups (*p* < 0.001). These associations are observational and should not be interpreted as independent treatment effects.

Kaplan–Meier analysis showed statistically significant differences in OS among the WHO CNS4 groups (*p* = 0.03) (Figure 3). After reassignment using the WHO CNS5-based study algorithm, differences among molecularly defined groups were not statistically significant (*p* = 0.13) (Figure 4). FISH-defined oligodendroglioma and IDHmut astrocytoma groups showed longer observed survival, but these estimates were based on small subgroups and should be interpreted cautiously. Female patients had longer OS than male patients in the unadjusted comparison (*p* = 0.02) (Figure 5a). There were statistically significant differences in OS among different age groups (*p* = 0.001) (Figure 5b). These analyses are exploratory and should not be interpreted as demonstrating that molecular reclassification changed prognosis.

The mean OS in the WHO CNS5-based groups was 33.3 ± 29.6 months for FISH-defined oligodendroglioma, 22.7 ± 29.8 months for astrocytoma, IDHmut, grades 2–3, 29.2 ± 23.5 months for astrocytoma, IDHmut, grade 4, 15.9 ± 8.9 months for astrocytoma, IDHwt, grades 2–3, and 22.9 ± 25.9 months for GBM, IDHwt (*p* = 0.13) (Table 2). Age stratification showed significant differences in OS (*p* < 0.001), with shorter observed survival among older patients. In multivariable Cox regression, age was the only independent predictor of OS according to the current analysis; molecular subtype, preoperative KPS, and EOR were not independently associated with outcome (Supplementary Materials, if applicable). Because the molecular subgroups were small and treatment occurred before reassignment, these survival estimates should be considered exploratory. The median OS for GBM, IDHwt was 16.1 months, whereas the median OS for FISH-defined oligodendroglioma was not reached during follow-up. Treatment-related variables were not sufficiently available or consistently documented across the cohort and were therefore not included in the survival analysis.

## 4. Discussion

This comparative analysis illustrates the difference between historical histologic categorization according to the WHO CNS4 [4] and the integrated molecular framework of WHO CNS5 [3]. In the present cohort, 51.5% of cases received a different diagnosis after molecular reassessment, particularly among tumors originally categorized as astrocytoma or glioma NOS. This diagnostic shift supports the value of molecular reassessment in settings where morphology alone may not fully resolve tumor identity. However, because the molecular workflow did not include comprehensive NGS or systematic +7/−10 testing, the magnitude of reclassification should not be interpreted as equivalent to complete WHO CNS5 molecular ascertainment.

Large-scale molecular datasets have demonstrated the utility of integrating genetic and epigenetic information for glioma classification [2,23,24,25]. These studies support the biological relevance of molecularly defined entities, but they also illustrate the limitations of applying a restricted marker panel to individual patients. Large-scale multi-omics datasets, including The Cancer Genome Atlas (TCGA), have reinforced the utility of the WHO CNS5 classification. Integration of curated molecular data enables updated diagnoses and improved risk stratification for most diffuse gliomas [24]. Multi-omics reclassification is feasible in 73.8% of TCGA glioma cases [25], while transcriptomic and DNA methylation profiling further support the biological validity of WHO CNS5-defined tumor entities. The present study contributes data from a Colombian cohort, a population that remains underrepresented in large molecular glioma datasets [15], but no inference regarding genetic ancestry can be made because ancestry was not directly assessed. The cohort should therefore be described geographically rather than as representing a molecularly defined Hispanic ancestry group.

The observed clinical and survival patterns should be interpreted in the context of small subgroup sizes and retrospective treatment. The WHO CNS5-based groups did not differ significantly in OS (*p* = 0.13), and the difference between *p* = 0.03 under the historical classification and *p* = 0.13 after reassignment should not be interpreted as evidence that WHO CNS5 reduced prognostic discrimination. Female sex was associated with longer OS in the unadjusted analysis (*p* = 0.02), whereas age was the only independent predictor in the reported multivariable model. The absence of a significant molecular-group survival difference does not establish equivalence between groups; confidence intervals and effect estimates should be emphasized in future validation cohorts. Treatment variables were not incorporated into the current adjusted analysis and may confound observed survival associations. Furthermore, the absence of statistically significant survival differences is likely related to the limited sample size and reduced statistical power. Our findings therefore support improved diagnostic and prognostic characterization rather than a demonstrated therapeutic or survival benefit. Survival estimates for several astrocytoma subgroups should be interpreted with caution due to very small sample sizes and extensive censoring. In multivariable Cox regression, age was the only independent predictor of OS, whereas molecular subtype, preoperative KPS, and EOR were not significantly associated with outcome after adjustment (Table S1 and Figure S1).

### 4.1. Protocols for Molecular Classification

Implementation of WHO CNS5 in LMICs requires balancing diagnostic completeness, cost, infrastructure, and turnaround time. A marker-based workflow can provide targeted information when NGS is not accessible, but it should be presented as a pragmatic strategy rather than as analytically equivalent to comprehensive sequencing. The workflow used in this study was based on IHC, FISH, PCR, and Sanger sequencing and should be interpreted within the limitations of each assay.

NGS can simultaneously detect single-nucleotide variants (e.g., IDH1/2 mutations), copy-number variations (e.g., 1p/19q codeletion, CDKN2A/B deletion), and gene fusions (e.g., MYB::QKI in angiocentric glioma) [16,26]. In contrast, marker-based methods provide targeted information and may be more accessible in resource-constrained laboratories. The choice between approaches depends on local infrastructure, specimen quality, turnaround requirements, and cost. In many clinical settings, complementary methods such as FISH, IHC, and qPCR remain essential, as they provide a marker-by-marker approach when NGS is not available or feasible. This is very common, as the cost of analyzing a single sample is significantly higher for NGS than for other methods [16]. Similar disparities apply to adjuvant glioma therapy: where insurance coverage exists, equitable and timely access to molecular testing or precision treatments is not guaranteed [27]. The present study did not perform a head-to-head analytical validation against NGS. Therefore, the observed diagnostic changes should not be interpreted as evidence that the marker-based approach has equivalent sensitivity or specificity.

Marker-by-marker approaches retain several advantages in glioma classification [16,18]. First, assays such as FISH, IHC, and qPCR are widely available, requiring less infrastructure and expertise than NGS, making them feasible in resource-limited environments. Second, they can support selective testing of high-yield biomarkers when budgets restrict comprehensive panels, although the relative economic implications depend on local costs and testing strategies. Third, they offer rapid turnaround, as exemplified by IDH1-R132H IHC, which serves as a fast and reliable screening tool. Fourth, targeted testing allows clinicians to prioritize markers based on patients’ age, tumor location, or histological features, with results from key biomarkers like IDH1 or 1p/19q codeletion directly informing prognosis and treatment planning. Finally, highly sensitive techniques, including digital droplet PCR and FISH, provide superior sensitivity for low-frequency variants or direct visualization of chromosomal aberrations within tumor cells, improving diagnostic precision even in samples with limited tumor purity [16,18].

In our institutional setting, the estimated cost of applying NGS to all patients, including sample preparation, sequencing, and bioinformatic analysis, was approximately USD 25,000 for the 68 cases. In comparison, our institution-specific estimated cumulative testing cost of the marker-by-marker diagnostic strategy was USD 11,250 across the same cohort, based on the individual immunohistochemical, FISH, and PCR-based tests performed according to the sequential diagnostic algorithm and as applicable to each case. Although the estimated direct testing expenditure was lower for the marker-by-marker approach in our setting, differences in direct testing costs may change as NGS becomes increasingly accessible [28]. These estimates are specific to our institutional context and were not intended to constitute a formal cost-effectiveness or microcosting analysis. Reagent and consumable costs were included in the institutional estimates; however, personnel costs, equipment depreciation, laboratory overhead, and costs associated with repeat testing were not systematically quantified.

### 4.2. Impact of Reclassification on Targeted Therapy

In clinical practice, comprehensive molecular profiling may provide greater breadth than single-marker assays, but analytical performance varies by assay and clinical context [29]. In gliomas, an IDH1 R132H IHC-negative result does not exclude noncanonical IDH1 or IDH2 mutations, and locus-specific FISH does not directly establish whole-arm 1p/19q codeletion [16]. These limitations are particularly relevant when a restricted molecular panel is used for WHO CNS5-based reassessment. Complementary sequencing can be considered when the available tests yield an ambiguous result or when a comprehensive molecular diagnosis is clinically required.

The practical value of a sequential workflow lies in targeted use of locally available assays and referral of diagnostically ambiguous cases for additional testing [30]. However, claims regarding cost savings, turnaround time, or clinical utility require direct measurement in prospective implementation studies. The present retrospective study did not evaluate these outcomes systematically. The molecular reassessment was performed after the patients had undergone their original treatment; therefore, this study cannot determine whether reclassification would have changed treatment selection, response, or survival. Future prospective studies should integrate standardized molecular testing before treatment with detailed radiotherapy, temozolomide, treatment-completion, and subsequent-therapy data.

### 4.3. Future Perspectives

Future studies should combine comprehensive molecular profiling with standardized clinical and treatment data. Prospective validation cohorts should include systematic IDH1/IDH2 sequencing in R132H-negative cases, robust assessment of whole-arm 1p/19q status, validated criteria for CDKN2A/B homozygous deletion, EGFR amplification thresholds, and assessment of +7/−10 where indicated. Integration of methylation profiling, copy-number analysis, spatial transcriptomics, and single-cell approaches may further refine classification and biological stratification [31,32].

Molecular reclassification of gliomas has refined diagnostic accuracy, improved risk stratification, and revealed previously unrecognized tumor entities [33]. Large-scale genomic studies continue to identify novel molecular subtypes and genetic markers associated with poor outcomes within specific tumor types [34]. Complementary epigenomic studies—such as differential microRNA expression and methylation profiling—further enhance subtype characterization and predictive modeling. These molecular insights improve clinicians’ ability to anticipate treatment response, optimize immunotherapy through a better understanding of glioma immune microenvironments, and explain the limited efficacy of immune checkpoint blockade [35]. Ultimately, integrating these approaches will facilitate the design of more homogeneous, biomarker-driven clinical trials and advance precision neuro-oncology [36]. Emerging technologies, including spatial transcriptomics and single-cell sequencing, are expected to further refine patient stratification and elucidate mechanisms of therapeutic resistance [31,32]. Expanding access to molecular testing and data-sharing initiatives across LMICs will be important for equitable implementation of precision neuro-oncology.

Finally, the molecular classification of IDHmut astrocytomas continues to evolve, with recent cIMPACT-NOW recommendations and emerging evidence suggesting that alterations involving CDK4, CDK6, CCND2, RB1, CDKN2A, PIK3CA/PIK3R1, EGFR, MYCN, and PDGFRA may further refine molecular grading and prognostic risk stratification beyond the current WHO CNS5 framework [37,38]. These findings may inform the next WHO classifications of CNS tumors, potentially enabling more precise molecular grading and risk stratification of IDHmut gliomas. Further validation in independent cohorts will be essential before their incorporation into routine classification.

### 4.4. Limitations of the Study

This study has several limitations. First, the cohort was modest in size (n = 68), with small WHO CNS5-based subgroups, limiting statistical power and the precision of survival estimates. Second, molecular reassessment was performed using a sequential, marker-based approach rather than comprehensive next-generation sequencing (NGS). In particular, R132H-negative tumors were not systematically screened for noncanonical IDH1 or IDH2 mutations, and +7/−10 status was not systematically assessed. Third, treatment-related variables, including radiotherapy, temozolomide, treatment completion, and subsequent therapies, were not systematically available and therefore could not be incorporated into adjusted survival analyses. Consequently, treatment-related confounding cannot be excluded. Fourth, molecular reassignment was performed retrospectively after treatment, precluding assessment of whether molecular classification would have altered therapeutic management.

Additional technical limitations included challenges in assessing EGFR amplification in paraffin-embedded tissue and the limited initial availability of CDKN2A/B FISH probes, which required completion of testing using the additional assays described in the Methods. These factors may have affected the completeness and timing of molecular reassessment.

The study also identified methodological gaps relevant to future implementation, including the need to systematically document assay completion and failure rates, turnaround time, tissue adequacy, and standardized costs. Future studies should address these parameters and validate sequential marker-based workflows against comprehensive molecular profiling in larger, independent cohorts. Accordingly, the survival analyses should be considered exploratory, and the molecular classifications should be interpreted as WHO CNS5-based assignments supported by the available testing rather than as comprehensive molecular diagnoses. Despite these limitations, our findings support the practical feasibility of a sequential molecular reassessment approach in this real-world clinical setting.

## 5. Conclusions

This study shows that sequential molecular reassessment using IHC, FISH, PCR, and Sanger sequencing can materially change the diagnostic categorization of adult diffuse gliomas in a Colombian cohort. However, the study should be interpreted as a feasibility-oriented diagnostic reassessment rather than as evidence of complete WHO CNS5 molecular classification or improved treatment outcomes. The diagnosis changed in 51.5% of cases, while survival differences among WHO CNS5-based groups were not statistically significant (*p* = 0.13). Age was the only independent predictor in the reported multivariable model, and female sex was associated with longer OS only in the unadjusted analysis. Because of small subgroups, incomplete molecular coverage, retrospective reassignment after treatment, and incomplete treatment data, these findings should not be used to infer treatment benefit or improved survival attributable to molecular reclassification. Prospective multicenter studies with comprehensive molecular testing and standardized treatment data are needed to determine the clinical consequences of WHO CNS5 implementation.

## Figures and Tables

**Table 1:** Clinical and Demographic Data. * Within the WHO CNS5-based study algorithm, oligodendroglioma was assigned to IDHmut tumors with the predefined 1p/19q FISH deletion pattern; because the assay was locus-specific, this pattern does not directly establish whole-arm 1p/19q codeletion.

Variable	Oligodendroglioma * (n = 8)	Astrocytoma, IDHmut Grade 2–3 (n = 9)	Astrocytoma, IDHmut Grade 4 (n = 4)	Astrocytoma, IDHwt (n = 2)	Glioblastoma (n = 45)	Total (n = 68)	*p* Value
Sex (number [%])							0.116
Male	5 (62.5%)	7 (77.8%)	4 (100.0%)	2 (100.0%)	22 (48.9%)	40 (58.8%)	
Female	3 (37.5%)	2 (22.2%)	0 (0.0%)	0 (0.0%)	23 (51.1%)	28 (41.2%)	
Age (mean ± SD)	44.8 ± 16.0	55.3 ± 13.7	63.8 ± 19.5	50.5 ± 2.1	58.6 ± 15.1	56.6 ± 15.7	0.246
Pathology—WHO CNS4. (number [%])							<0.001
Oligodendroglioma	5 (62.5%)	3 (33.3%)	0 (0.0%)	N/A	4 (8.9%)	12 (17.6%)	
Astrocytoma	1 (12.5%)	3 (33.3%)	N/A	2 (100.0%)	9 (20.0%)	15 (22.1%)	
Glioma NOS	1 (12.5%)	3 (33.3%)	N/A	N/A	4 (8.9%)	8 (11.8%)	
Glioblastoma	1 (12.5%)	N/A	4 (100.0%)	N/A	28 (62.2%)	33 (48.5%)	
Location (number [%])							0.971
Frontal	3 (37.5%)	4 (44.4%)	1 (25.0%)	1 (50.0%)	12 (26.7%)	21 (30.9%)	
Frontoparietal	0 (0.0%)	1 (11.1%)	1 (25.0%)	0 (0.0%)	2 (4.4%)	4 (5.9%)	
Parietal	1 (12.5%)	0 (0.0%)	0 (0.0%)	0 (0.0%)	5 (11.1%)	6 (8.8%)	
Insular	2 (25.0%)	1 (11.1%)	0 (0.0%)	0 (0.0%)	3 (6.7%)	7 (10.3%)	
Basal Ganglia	0 (0.0%)	0 (0.0%)	0 (0.0%)	0 (0.0%)	2 (4.4%)	2 (2.9%)	
Temporal	2 (25.0%)	2 (22.2%)	1 (25.0%)	1 (50.0%)	11 (24.4%)	17 (25.0%)	
Posterior fossa	0 (0.0%)	0 (0.0%)	0 (0.0%)	0 (0.0%)	4 (8.9%)	4 (5.9%)	
Bilateral	0 (0.0%)	1 (11.1%)	0 (0.0%)	0 (0.0%)	1 (2.2%)	3 (4.4%)	
Occipital	0 (0.0%)	0 (0.0%)	1 (25.0%)	0 (0.0%)	4 (8.9%)	5 (7.4%)	
Patients’ laterality (number [%])							0.314
Left sided	2 (25.0%)	0 (0.0%)	0 (0.0%)	0 (0.0%)	4 (8.8%)	8 (11.8%)	
Right sided	6 (75.0%)	9 (100.0%)	4 (100.0%)	2 (100.0%)	41 (91.1%)	60 (88.2%)	
Hemisphere compromised (number [%])							0.794
Left	3 (37.5%)	3 (33.3%)	2 (50.0%)	2 (100.0%)	24 (53.3%)	34 (50.0%)	
Right	4 (50.0%)	5 (55.6%)	2 (50.0%)	0 (0.0%)	19 (42.2%)	30 (44.1%)	
Midline or infratentorial	1 (12.5%)	1 (11.1%)	0 (0.0%)	0 (0.0%)	2 (4.4%)	4 (5.9%)	
Bilateral	0 (0.0%)	0 (0.0%)	0 (0.0%)	0 (0.0%)	0 (0.0%)	0 (0.0%)	
Presenting symptom (number [%])							0.207
Seizures	4 (50.0%)	1 (11.1%)	0 (0.0%)	1 (50.0%)	9 (20.0%)	15 (22.1%)	
Motor deficit	4 (50.0%)	4 (44.4%)	4 (100.0%)	1 (50.0%)	15 (33.3%)	28 (41.2%)	
Language deficit	2 (25.0%)	4 (44.4%)	4 (100.0%)	1 (50.0%)	10 (22.2%)	21 (30.9%)	
Sensitive deficit	1 (12.5%)	3 (33.3%)	4 (100.0%)	0 (0.0%)	22 (48.9%)	30 (44.1%)	
Behavior impairment	4 (50.0%)	0 (0.0%)	0 (0.0%)	1 (50.0%)	19 (42.2%)	24 (35.3%)	
Preoperative KPS (mean ± SD)	95.7 ± 5.3	85.0 ± 15.2	82.5 ± 15.0	95.0 ± 7.1	92.0 ± 14.2	91.2 ± 13.5	0.306
Postoperative KPS (mean ± SD)	94.3 ± 5.3	65.0 ± 23.5	70.0 ± 23.1	75.0 ± 35.4	86.2 ± 13.1	83.6 ± 16.7	0.036
Follow-up in months (mean ± SD)	33.3 ± 29.6	22.7 ± 31.6	29.2 ± 27.1	15.9 ± 12.6	22.9 ± 26.2	24.4 ± 26.7	0.637
Extent of resection (number [%])							0.217
GTR	1 (12.5%)	2 (22.2%)	0 (0.0%)	0 (0.0%)	5 (11.1%)	8 (11.8%)	
NTR	4 (50.0%)	5 (55.6%)	4 (100.0%)	1 (50.0%)	13 (28.9%)	27 (39.7%)	
STR	2 (25.0%)	1 (11.1%)	0 (0.0%)	1 (50.0%)	26 (57.8%)	30 (44.1%)	
Biopsy	1 (12.5%)	1 (11.1%)	0 (0.0%)	0 (0.0%)	1 (2.2%)	3 (4.4%)	
New onset postoperative seizures (number [%])	1 (12.5%)	4 (44.4%)	4 (100.0%)	1 (50.0%)	10 (22.2%)	20 (29.4%)	0.010
New onset language deficit (number [%])	1 (12.5%)	4 (44.4%)	0 (0.0%)	2 (100.0%)	9 (20.0%)	16 (23.5%)	0.029
New onset motor deficit (number [%])	1 (12.5%)	1 (11.1%)	0 (0.0%)	2 (100.0%)	14 (31.1%)	18 (26.5%)	0.052

Abbreviations: Oligodendroglioma * = FISH-defined oligodendroglioma; AST = Astrocytoma; IDHmut = IDH mutant; IDHwt = IDH wildtype; GBM = Glioblastoma; KPS = Karnofsky Performance Scale; GTR = Gross Total Resection; NTR = Near Total Resection; STR = Subtotal Resection. *p* values: Kruskal–Wallis test for continuous variables; chi-square or Fisher’s exact test for categorical variables. N/A = not available. KPS was recorded in 10-point increments and is reported on the 0–100 scale; values of 0 and 5 were treated as missing-data codes rather than scores, as a preoperative KPS of 0 is incompatible with the corresponding postoperative values. Comparison was not performed owing to insufficient data.

**Table 2:** Diagnostic reassignment and overall survival according to the historical histologic classification (WHO CNS4) and the WHO CNS5-based study algorithm. Overall survival is expressed as mean ± SD, with median survival estimated by Kaplan–Meier methods.

Classification Criteria	Tumor Subtype (n)	Overall Survival (Months, Mean ± SD)	Median Overall Survival (Months) (IQR: Q1–Q3) †
WHO CNS4-based study algorithm			
	Oligodendroglioma (n = 12)	39.2 ± 34.0	NR (NR-NR)
	Astrocytoma (n = 15)	18.4 ± 23.7	13.5 (7.0-NR)
	Glioma, NOS (n = 8)	24.0 ± 25.7	NR (2.3-NR)
	Glioblastoma (n = 33)	21.5 ± 22.5	12.0 (4.0–58.1)
WHO CNS5-based study algorithm			
	Oligodendroglioma (IDHmut, FISH-defined 1p/19q deletion pattern) (n = 8)	33.3 ± 27.7	NR (NR-NR)
	Astrocytoma IDHmut grades 2–3 (n = 9)	22.7 ± 29.8	8.6 (5.4-NR)
	Astrocytoma IDHmut grade 4 (n = 4)	29.2 ± 23.5	11.9 (11.9-NR)
	Astrocytoma IDH wildtype (n = 2)	15.9 ± 8.9	7.0 (7.0–24.9)
	Glioblastoma IDH wildtype (n = 45)	22.9 ± 25.9	16.1 (4.6-NR)

OS = Overall Survival; IQR = Interquartile Range; Q1 = 1st quartile (time at S(t) = 0.75); Q3 = 3rd quartile (time at S(t) = 0.25); IDHmut = IDH mutant; IDHwt = IDH wildtype; Codel. = codeletion; SD = standard deviation. † Median OS and interquartile range (IQR) were estimated using the Kaplan–Meier method. “NR” (not reached) indicates that the corresponding quartile was not reached during follow-up (i.e., ≥50% or ≥75% of patients remained alive). Lower bound value: because the median was not reached, the reported value represents the last observed time point at which the estimated survival probability, S(t), was ≥0.50.

## Data Availability

Additional data are available upon reasonable request from the corresponding author.

## Supplementary Materials

The following supporting information can be downloaded at: https://www.mdpi.com/article/10.3390/cancers18203364/s1, Table S1: Multivariable Cox proportional hazards regression analysis of overall survival. The model assessed the independent association of age, preoperative Karnofsky Performance Status (KPS), extent of resection, and WHO CNS5 molecular subtype with overall survival. Hazard ratios (HRs), 95% confidence intervals (CIs), and p values are presented, using IDH-wildtype glioblastoma as the reference category. Age was the only variable independently associated with overall survival; Figure S1: Multivariable Cox proportional hazards regression analysis of overall survival. Forest plot showing hazard ratios (HRs) and 95% confidence intervals (95% CIs) derived from a multivariable Cox proportional hazards regression model evaluating independent predictors of overall survival. The model was adjusted for age, preoperative Karnofsky Performance Status (KPS), extent of resection (gross total resection [GTR] vs. non-GTR), and molecular subtype according to the WHOCNS5 classification, using IDH-wildtype glioblastoma (GBM IDH-wildtype) as the reference category. HRs < 1 indicate a reduced risk of death, whereas HRs > 1 indicate an increased risk of death. Age was the only variable independently associated with overall survival (HR = 1.05, 95% CI: 1.02–1.07; *p* < 0.001), while preoperative KPS, extent of resection, and WHOCNS5 molecular subtype were not independently associated with overall survival after multivariable adjustment.

## Abbreviations

The following abbreviations are used in this manuscript:

IDH1	Isocitrate dehydrogenase 1
ATRX	Alpha-thalassemia/mental retardation, X-linked
TERT	Telomerase reverse transcriptase
EGFR	Epidermal growth factor receptor
OS	Overall survival
+Ch7/−Ch10	Concurrent gain of chromosome 7 and loss of chromosome 10
WHO	World Health Organization
TCGA	The cancer genome atlas
LMICs	Low-to-middle-income countries
NGS	Next-generation sequencing
qPCR	Quantitative polymerase chain reaction
FISH	Fluorescence in situ hybridization
KPS	Karnofsky Performance Score
EOR	Extent of resection
LGG	Low-grade glioma
GTR	Gross-total resection
STR	Subtotal resection
NTR	Near-total resection
GBM	Glioblastoma
R132	Codon 132
R132H	Histidine substitution in codon 132
ICH	Immunochemistry
FFPE	Formalin-fixed paraffin-processed
CNS	Central nervous system
pTERT	Telomerase reverse transcriptase promoter
DNA	Deoxyribonucleic acid
WHO CNS4	4th edition of the world health organization classification of tumors of the central nervous system
MSR	Maximal safe resection
HGG	High-grade glioma
WHO CNS5	5th edition of the world health organization classification of tumors of the central nervous system
