# Supplementary material for: Molecular Reclassification of Diffuse Gliomas: A Feasibility Study of Clinical Features and Survival Outcomes in Colombia
Source: Cancers (Basel). 2026 Oct 17;18(20):3364. doi: 10.3390/cancers18203364 (PMC13702763; doi:10.3390/cancers18203364)
Supplement: Supplementary file 1 [file cancers-18-03364-s001.zip › cancers-4555187-supplementary.pdf]

**Supplementary Materials:**

**Table S1: Multivariable Cox proportional hazards regression analysis of overall survival.** The model assessed the independent association of age, preoperative Karnofsky Performance Status (KPS), extent of resection, and WHO CNS5 molecular subtype with overall survival. Hazard ratios (HRs), 95% confidence intervals (CIs), and p values are presented, using IDH-wildtype glioblastoma as the reference category. Age was the only variable independently associated with overall survival.

| N = 68 patients · 36 events · Reference: GMB IDH wildtype (n = 45) | | | | |
|---|---|---|---|---|
| **Variable** | **HR** | **95% CI (lower)** | **95% CI (upper)** | **P value** |
| **GBM IDH wt (reference)** | — | — | — | — |
| **Clinical variables** | | | | |
| **Age (per year)** | **1.05** | 1.02 | 1.07 | **<0.001** |
| **Preoperative KPS (per point)** | 1.00 | 0.99 | 1.02 | 0.536 |
| **Extent of resection: GTR vs non-GTR** | 0.44 | 0.08 | 2.58 | 0.367 |
| **WHOCNS5 molecular subtype** | | | | |
| **Oligodendroglioma IDH mutant vs GBM IDHwt** | 0.27 | 0.06 | 1.30 | 0.103 |
| **Astrocytoma IDH mutant grade 2–3 vs GBM IDHwt** | 1.30 | 0.48 | 3.52 | 0.603 |
| **Astrocytoma IDH mutant grade 4 vs GBM IDHwt** | 0.63 | 0.18 | 2.28 | 0.485 |
| **Astrocytoma IDH wildtype vs GBM IDHwt** | 2.36 | 0.55 | 10.14 | 0.250 |

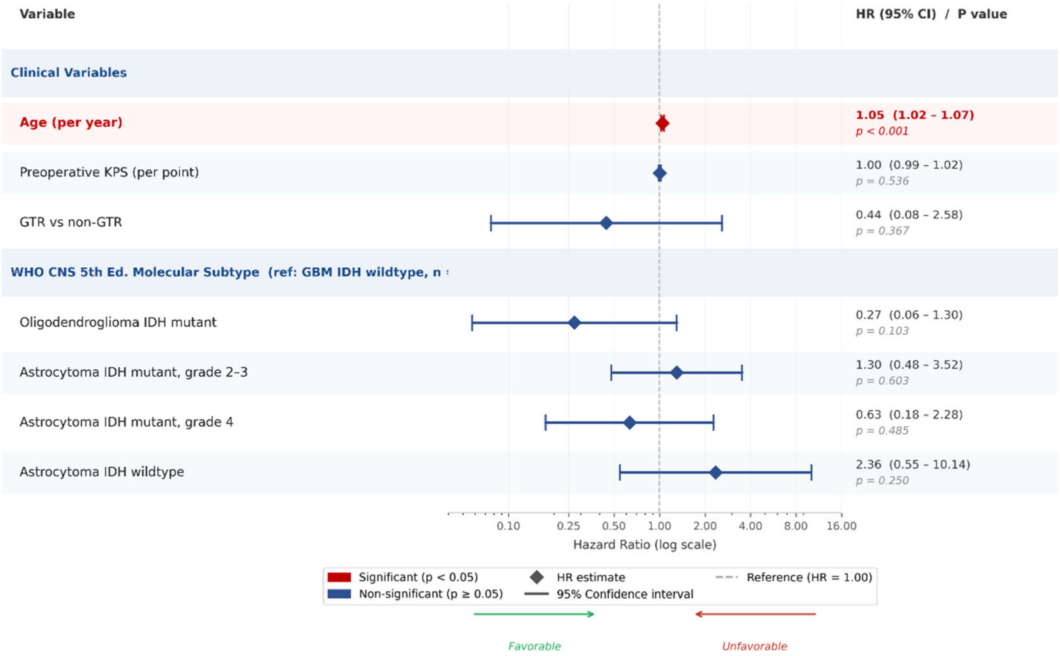


**Figure S1: Multivariable Cox proportional hazards regression analysis of overall survival.** Forest plot showing hazard ratios (HRs) and 95% confidence intervals (95% CIs) derived from a multivariable Cox proportional hazards regression model evaluating independent predictors of overall survival. The model was adjusted for age, preoperative Karnofsky Performance Status (KPS), extent of resection (gross total resection [GTR] vs. non-GTR), and molecular subtype according to the WHOCNS5 classification, using IDH-wildtype glioblastoma (GBM IDH-wildtype) as the reference category. HRs <1 indicate a reduced risk of death, whereas HRs >1 indicate an increased risk of death. Age was the only variable independently associated with overall survival (HR = 1.05, 95% CI: 1.02–1.07; $p < 0.001$), while preoperative KPS, extent of resection, and WHOCNS5 molecular subtype were not independently associated with overall survival after multivariable adjustment.
